# Short-Term Outcomes of COVID-19 Pandemic on Non-Small Cell Lung Cancer Screening and Management

**DOI:** 10.1055/s-0043-1777856

**Published:** 2023-12-20

**Authors:** Felix Orelaru, Melanie Edwards, Ruth Raleigh, Ali Abunayla, Rachel Bush, Shannon Porter, Kate Schumaker, Jeremy Albright, Kumari N. Adams

**Affiliations:** 1 Division of Cardiothoracic Surgery, Trinity Health Ann Arbor Hospital, Ypsilanti, Michigan

**Keywords:** COVID-19, non-small cell lung cancer, radiotherapy, surgical management

## Abstract

**Background** To assess the impact of coronavirus disease 2019 (COVID-19) pandemic on non-small cell lung cancer (NSCLC) screening, staging, and management in a single health care system.

**Materials and Methods** From November 2015 to December 2020, a total of 1,547 NSCLC cases was reported at our institution including 1,329 cases pre-COVID-19 and 218 cases during COVID-19. Pre-COVID-19 was defined as November 2015 to February 2020, while during COVID-19 was March 2020 to December 2020. Data were collected from tumor registry and medical record review. Patients with mesothelioma, lymphoma, small cell, or mixed small cell cancer were excluded from the study.

**Results** Both pre-COVID-19 and during COVID-19 cohorts had similar comorbidities including age (70 vs. 71 years), current smokers (35 vs. 32%), and chronic obstructive lung disease (32 vs. 28%). The number of low-dose computed tomography lung cancer screening scans decreased by 25% during COVID-19 compared with pre-COVID-19 era. There were more cases of stage 1A NSCLC pre-COVID-19 (31 vs. 25%) and more stage 4 cancer during COVID-19 (42 vs. 33%); *p* = 0.01. The proportion of patients treated with radiotherapy was similar between pre-COVID-19 and during COVID-19 (49 vs. 50%), but fewer patients underwent surgery during COVID-19 (17 vs. 27%; *p* = 0.004). The median time to radiotherapy (67 days) and surgery (29 days) was similar between the groups. The unadjusted overall 6-month mortality after lung cancer diagnoses was higher during COVID-19 compared with pre-COVID-19 (28 vs. 22%; *p* = 0.04).

**Conclusion** The COVID-19 pandemic resulted in delayed lung cancer screening scans, and more patients had diagnosis of advanced NSCLC; however, short-term mortality was unchanged.

Lung cancer is the leading cause of cancer-related deaths worldwide, accounting for approximately 1.6 million deaths per year, 1 and survival is strongly correlated to cancer stage at the time of diagnosis. 2 The national lung screening trial (NLST) identified that early screening with low-dose computed tomography (CT) scans decrease lung cancer mortality by 20%. 3 Furthermore, literature shows that early screening, diagnosis, and surgical management of non-small cell lung cancer (NSCLC) improve overall survival outcomes. 4 5 However, the coronavirus disease 2019 (COVID-19) pandemic disrupted routine lung cancer screening and management. COVID-19 was declared a pandemic in March 2020 as a result of human infection with severe acute respiratory syndrome coronavirus 2 (SARS-CoV-2), also known as COVID-19 virus, leading to severe respiratory illness. At the peak of COVID-19 pandemic, there was a universal delay in lung cancer screening, and a shift toward nonsurgical treatment of early-staged NSCLC such as stereotactic ablative radiotherapy (SABR) as an alternative method to potentially mitigate exposure risks to COVID-19 and at some institutions as a bridge to delayed surgical resection of NSCLC. 6 Many hospitals across the United States significantly reduced their operative volume to preserve personal protective equipment, reduce COVID-19 viral transmission, and decrease health care burden. The short- and long-term impact of delayed low-dose CT lung cancer screening scans and surgical treatment of NSCLC during the COVID-19 pandemic are not well known. This study aims to assess the short-term outcome of COVID-19 pandemic on NSCLC screening, staging, and management including radiotherapy versus surgical resection, in a single health care system in order to help guide future NSCLC management guidelines.

## Materials and Methods

This study was approved by the Institutional Review Board at Trinity Health Ann Arbor Hospital (E-21-977; October 13, 2021) and a waiver of informed consent was obtained.

### Study Population

Between November 23, 2015, and December 28, 2020, a total of 1,547 NSCLC cases was reported at Trinity Health Ann Arbor, Chelsea, and Livingston hospitals. Of this population, 1,329 cases were reported pre-COVID-19 and 218 cases during COVID-19. Pre-COVID-19 was defined as November 2015 to February 2020, while during COVID-19 was March 2020 to December 2020. All patients ≥ 18 years old, who had a new histologic diagnosis of NSCLC at our institution were included in the study. Patients who had a new NSCLC diagnosis at outside institution but presented to our hospital for treatment were also included in the study. Patients with mesothelioma, lymphoma, small cell, or mixed small cell cancer were excluded from the study.

Patient comorbidities, lung cancer screening status, cancer stage at diagnosis, treatment modality, and mortality were collected from tumor registry at Trinity Health System—Ann Arbor, Chelsea, and Livingston hospitals, and supplemented with medical record review. Primary outcomes were defined as proportion of NSCLC low-dose CT screening scans, NSCLC diagnosis, and treatment including surgical resection or SABR, during COVID-19 pandemic compared with pre-COVID-19 era. Secondary outcomes included time from NSCLC diagnosis to SABR versus surgical resection.

### Statistical Analysis

Data were presented as median (25, 75%) for continuous data and *n* (%) for categorical data. Patient demographics and comorbidities were presented by exposure. The distribution of clinical NSCLC stages pre-COVID-19 and during COVID-19 were compared using both Mann–Whitney's tests and a Fisher's exact test. Time from diagnosis to SABR and time from diagnosis to surgical resection are summarized with medians and interquartile ranges and tested with Mann–Whitney's test. Chi-square test was used to compare radiation and surgery rates. Mortality was treated as binary, rather than assessed as time to the event, given that the pre-COVID-19 sample had longer follow-up. Death was measured as mortality within 30, 180, and 365 days from NSCLC diagnosis. A likelihood ratio test comparing logistic regression models with and without NSCLC stage by period was also performed. Time to radiotherapy and surgery were calculated from the date of NSCLC diagnosis, that is, the date the patient was first diagnosed with a reportable NSCLC cancer on imaging, physician statement of suspicious cancer, or histologically.

## Results

**Fig. 1:**
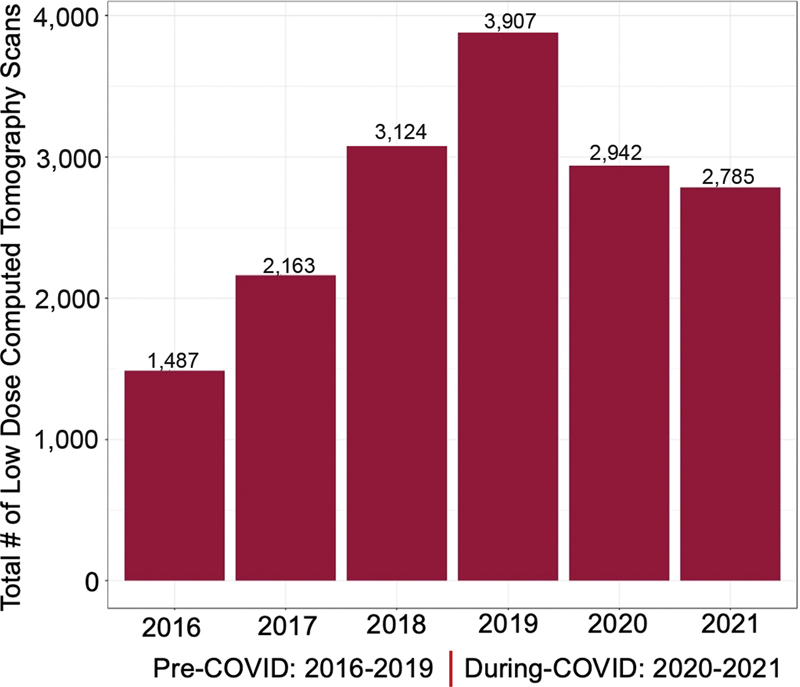
There was an increasing trend in number of routine low-dose computed tomography scans performed by year from 2016 through 2019 (pre-COVID-19) followed by a 25% drop in 2020 (during COVID-19) that continues through 2021.

**Fig. 2:**
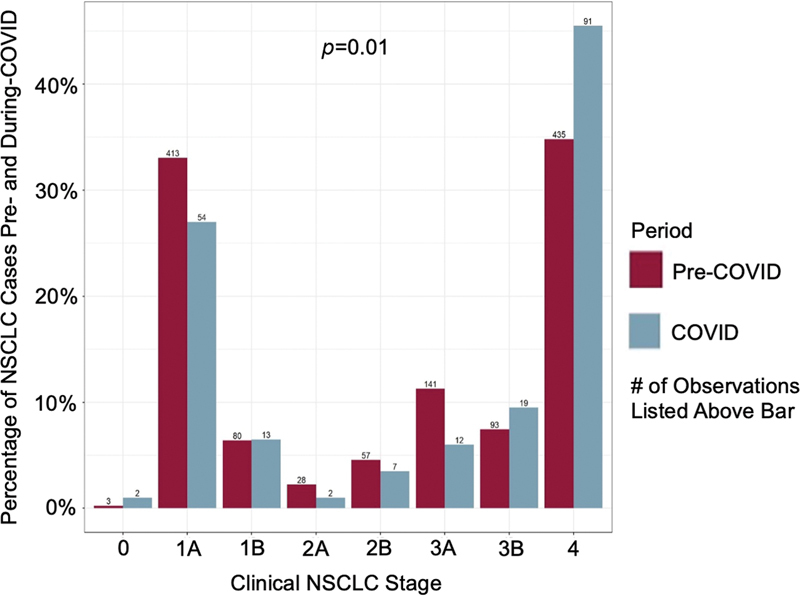
Distribution of non-small cell lung cancer (NSCLC) clinical stages by period. The percentage of patients with stage 1A cancers were more common in the pre-COVID-19 period, while the number of stage 4 cancers were more common in the during COVID-19 period. On an ordinal scale, the median clinical stage in the pre-COVID-19 period was 3A, and 3B in the COVID-19 period, *p* = 0.01.

**Fig. 3:**
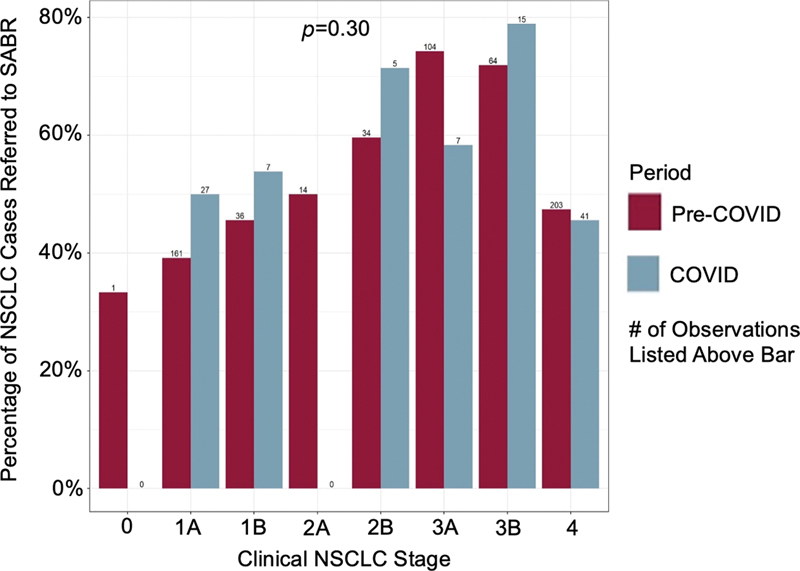
There was no significant difference in radiotherapy referral rates of non-small cell lung cancer (NSCLC) clinical stage-by-period. *p* = 0.30.

**Fig. 4:**
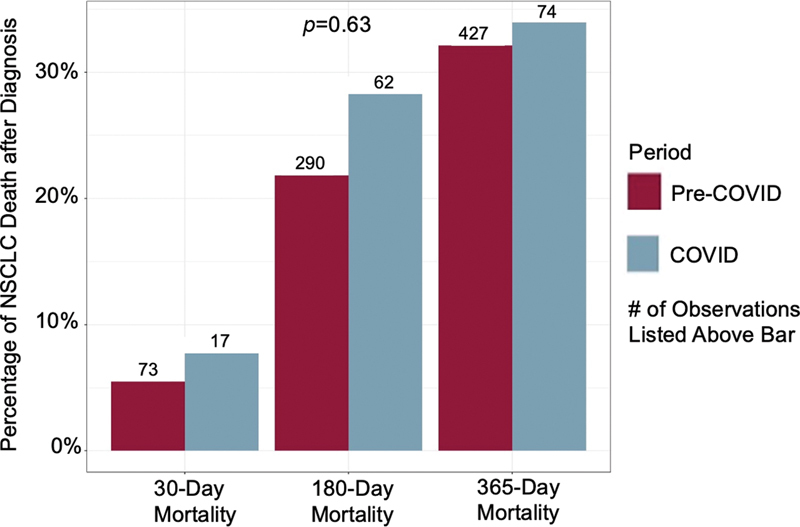
The 30-day, 6-month, and 1-year mortality rates following non-small cell lung cancer (NSCLC) diagnosis by period. Unadjusted 180-day mortality was significantly higher during COVID-19 period, chi-square test (1) = 4.3, *p* = 0.04, but not significant after adjusting for demographics and comorbidities ( *p* = 0.63). Unadjusted and adjusted 30- and 365-day mortalities were not significantly different between periods.

Both pre-COVID-19 and during COVID-19 cohorts had similar demographic data and comorbidities including age (70 vs. 71 years; *p* = 0.17), male sex (48 vs. 48%; *p* = 0.97), current smokers (35 vs. 32%; *p* = 0.13), diabetes (13 vs. 14%; *p* = 0.73), chronic obstructive pulmonary disease (32 vs. 28%; *p* = 0.23), chronic kidney disease (7 vs. 5%; *p* = 0.24), and stroke (15 vs. 12%; *p* = 0.22) ( Table 1 ). In pre-COVID-19, the number of yearly routine low-dose CT screening scans increased by 20 to 30% compared with during COVID-19 where it decreased by 25% ( Fig. 1 ). There were more cases of stage 4 NSCLC during COVID-19 (42 vs. 33%; *p* = 0.01) ( Fig. 2 ), but the proportion of NSCLC cases by clinical stage referred for radiotherapy was similar to pre-COVID-19 era; *p* = 0.30 ( Fig. 3 ). Furthermore, fewer patients underwent surgical resection during COVID-19 (17 vs. 27%; *p* = 0.004) ( Table 2 ). The median time to radiotherapy (67 vs. 67 days, *p* = 0.82) and surgery (30 vs. 29 days, *p* = 0.64) were similar during COVID-19 compared with pre-COVID-19 ( Table 2 ). There was no significant difference in 1-year mortality among all NSCLC cases that underwent radiotherapy pre-COVID-19 compared with during COVID-19 (17 vs. 12%, *p* = 0.1). Also, the 1-year mortality rate was similar among NSCLC cases that underwent surgical resection pre-COVID-19 and during COVID-19 (2.03 vs. 0.46%, *p* = 0.17). However, the unadjusted overall 6-month mortality after lung cancer diagnoses (all stages of NSCLC) was higher during COVID-19 compared with pre-COVID-19 (28 vs. 22%; *p* = 0.04). After adjusting for demographics and comorbidities, the overall 6-month and 1-year mortalities after lung cancer diagnoses (all stages of NSCLC) were similar during COVID-19 compared with pre-COVID-19 ( *p* = 0.63) ( Fig. 4 ).

**Table 1:** Demographics and preexisting conditions of non-small cell lung cancer cases pre-COVID-19 and during COVID-19

Variable	Overall	Pre-COVID-19	During COVID-19	*p* -Value
Age (y)	70.09 (10.34)	69.95 (10.26)	70.99 (10.82)	0.168
Sex (male)	740 (47.83%)	635 (47.78%)	105 (48.17%)	0.974
Race				
White	1,432 (92.57%)	1,234 (92.85%)	198 (90.83%)	0.221
Black	97 (6.27%)	82 (6.17%)	15 (6.88%)	
Other	18 (1.16%)	13 (0.98%)	5 (2.29%)	
Smoking status				
Current	531 (34.35%)	462 (34.79%)	69 (31.65%)	0.128
Former	866 (56.02%)	746 (56.17%)	120 (55.05%)	
Nonsmoker	149 (9.64%)	120 (9.04%)	29 (13.3%)	
Hypertension	601 (38.85%)	527 (39.65%)	74 (33.94%)	0.127
Diabetes	205 (13.25%)	174 (13.09%)	31 (14.22%)	0.728
COPD	484 (31.29%)	424 (31.9%)	60 (27.52%)	0.225
Asthma	50 (3.23%)	40 (3.01%)	10 (4.59%)	0.311
Pneumonia	123 (7.95%)	107 (8.05%)	16 (7.34%)	0.822
CKD	103 (6.66%)	93 (7%)	10 (4.59%)	0.239
Stroke	231 (14.93%)	205 (15.43%)	26 (11.93%)	0.215

Abbreviations: CKD, chronic kidney disease; COPD, chronic obstructive disease.

Notes: Data presented as median (25, 75%) for continuous data and proportion (%) for categorical data. The *p* -value <0.05 indicates statistically significant difference between pre-COVID-19 and during COVID-19 groups.

**Table 2:** Treatment modality and time from NSCLC diagnosis to treatment

Variable	*N*	Overall	Pre-COVID-19	During COVID-19	*p* -Value
Radiation (SABR)	1,530	751 (49.08%)	644 (49.01%)	107 (49.54%)	0.944
Surgical resection	1,547	397 (25.66%)	359 (27.01%)	38 (17.43%)	0.004
Median time from diagnosis to radiation (d)	746	67 (41, 93)	67 (41, 93)	67 (45, 92)	0.817
Median time from diagnosis to surgery (d)	390	29 (0, 53)	29 (0, 53)	29.5 (12.5, 52)	0.641

Abbreviations: NSCLC, non-small cell lung cancer; SABR, stereotactic ablative radiotherapy.

Notes: Data presented as median (25, 75%) for continuous data and proportion (%) for categorical data. The *p* -value <0.05 indicates statistically significant difference between pre-COVID-19 and during COVID-19 groups.

## Discussion

In this study, we identified that the number of routine low-dose CT lung screening scans performed during COVID-19 decreased sharply compared with pre-COVID-19 era. Also, there were more cases of advanced (stage 4) NSCLC diagnosed during COVID-19. The median time to radiotherapy or surgical resection was similar pre- and during COVID-19. But the proportion of patients treated with radiotherapy was higher during COVID-19 and fewer patients underwent surgical resection compared with pre-COVID-19. The unadjusted 6-month mortality after lung cancer diagnoses was higher during the identified COVID-19 period but similar 6-month and 1-year mortality after adjusting for demographics and comorbidities compared with pre-COVID-19.

At the peak of the COVID-19 pandemic, many hospitals across the United States deferred routine health care screening and reduced their operative volume favoring only emergent surgeries in order to reduce COVID-19 virus exposure and transmission among patients and health care workers, preserve personal protective equipment, and decrease health care burden. This approach contributed to delayed routine lung cancer screening low-dose CT scans and a shift toward nonsurgical treatment of NSCLC, that is, SABR during the COVID-19 pandemic. 6 However, there is conflicting evidence in literature on the short- and long-term impact of delayed NSCLC screening and surgical management, especially during the COVID-19 pandemic. Earlier studies reported that delayed surgical resection of stage 1 NSCLC by 6 to 8 weeks from diagnosis was associated with higher likelihood of cancer upstaging and worsened long-term survival outcome. 4 7 But other studies show that surgical delay of stage 1 NSCLC resection by up to 12 weeks from diagnosis was not associated with pathologic upstaging, though patients had decrease survival rates and increased risk of recurrence. 8

In this study, we identified that in the pre-COVID-19 era, the number of yearly routine low-dose CT screening scans increased yearly by 20 to 30%, but during COVID-19, it decreased significantly by 25%. This could be due to high health care burden at the peak of the pandemic and a shift toward emergent imaging studies and postponing routine elective screening examinations, or patients' preference to limit health care interaction due to stay-at-home public health policies. Similar to our study, Patt et al (2020) reported that lung cancer screening decreased by 56% during COVID-19 pandemic (March–July 2020). 9 Kasymjanova et al (2021) also showed that the number of lung cancer diagnoses decreased by 21% during the pandemic (March 2020–February 2021). 10 This trend is worrisome and could contribute to delayed diagnosis and poor prognosis of NSCLC patients. The NLST identified that early lung cancer screening with low-dose CT scans decrease mortality by 20% compared with radiography. 3 Further evidence suggest that early NSCLC screening and diagnosis is associated with improved survival. 11 12

In addition, in our study, we identified more cases of stage IV NSCLC during COVID-19 compared with early-staged NSCLC (stages 1A and 3A) in pre-COVID-19 era. Similarly, Cantini et al (2022) showed that newly diagnosed lung cancer during COVID-19 (March–December 2020) were 5% more likely to be stage 4 cancers compared with pre-COVID-19 (March–December 2019). 13 It is therefore possible that NSCLC patients had upstaging of their lung cancer due to delayed screening/diagnosis during COVID-19. Our study discovery was confirmed by Mynard et al (2022) who reported a significant increase in stage 4 NSCLC diagnoses after a 11-week COVID-19 lockdown period in New York. 14 Furthermore, in our health care system, the proportion of NSCLC cases treated with radiotherapy was similar during COVID-19 compared with pre-COVID-19, but fewer patients underwent surgical resection during COVID-19. Kasymjanova et al (2021) reported a similar significant decrease in surgical resection of lung cancer during the pandemic but threefold increase utilization of radiotherapy and delayed surgery (>10 days) relative to pre-COVID-19 era. 10 We also observed that the median time to surgery or radiotherapy were similar during COVID-19 versus pre-COVID-19 era. But this could be because at our institution, we follow NSCLC patients closely and utilize a multidisciplinary team approach, ensuring they receive appropriate treatment as early as possible, in line with our hospital policies and guidelines during the pandemic. However, as reported in our study, fewer patients underwent surgical resection during the COVID-19 pandemic, although the time to surgery was unchanged.

Finally, the unadjusted overall 6-month mortality after lung cancer diagnoses was 6% higher during COVID-19 compared with pre-COVID-19 (28 vs. 22%). However, after adjusting for demographics and comorbidities, the overall 6-month and 1-year mortalities were similar. In addition, during COVID-19, the 1-year mortality among NSCLC cases after radiotherapy was higher (12%) compared with cases that underwent surgical resection (0.46%). Similar to our study, Garassino reported a high mortality rate (33%) among lung cancer patients infected with COVID-19. 15 Kabarriti et al (2020) demonstrated that lung cancer patients treated with radiotherapy 1 to 12 months prior to COVID-19 infection had threefold increased risk of death. 15 They also reported a linear correlation between lung radiation therapy dose and mortality risk after COVID-19 diagnosis in lung cancer patients. 16 It is possible that the underlying NSCLC in COVID-19 patients exacerbated SARS-CoV-2 virus-induced severe lung inflammation and parenchymal damage that led to worsened patient mortality.

Our study was a single health care system retrospective analysis and may not be generalizable to other hospitals across the United States. The observed difference in proportion of lung cancer screening CT scans pre-COVID-19 compared with during COVID-19 may not be solely related to the COVID-19 pandemic; however, other reported studies support our findings. 9 10 Finally, the higher proportion of stage 4 NSCLC during COVID-19 pandemic may have been because of more advanced staged cancer patients presented to the hospital due to worsened respiratory symptoms. However, other studies have confirmed our findings of cancer upstaging. The during COVID-19 study period of approximately 40 weeks (March–December 2020) is ample time for NSCLC disease progression if patients do not receive proper treatment as a result of decreased access to care at the peak of the COVID-19 pandemic. Literature shows that the volume-doubling time of squamous cell carcinoma is about 21 weeks. 17

## Conclusion

In conclusion, our study showed that the COVID-19 pandemic impacted the use of routine low-dose CT lung cancer screening scans and more patients had metastatic NSCLC at the time of diagnosis during COVID-19 pandemic. The unadjusted short-term mortality among NSCLC patients was higher during COVID-19 pandemic but similar to pre-COVID-19 era after adjusting for comorbidities. To date, lung cancer is the leading cause of cancer-related deaths globally. Concerted efforts to improve lung cancer screening can lead to increased diagnosis of early-staged NSCLC, subsequent treatment, and reduced mortality.
